# Can the Length of the Tibia Nail Be Predicted Correctly Before the Operation According to the Patient’s Height and Shoe Size?

**DOI:** 10.7759/cureus.52653

**Published:** 2024-01-21

**Authors:** Rıfat Şahin, Suat Şahin, Cengiz Kazdal, Mehmet Sabri Balık

**Affiliations:** 1 Orthopaedics and Traumatology, Recep Tayyip Erdogan University/Faculty of Medicine, Rize, TUR

**Keywords:** preoperative planning, tibial fracture, intramedullary nail, shoe size, body height, tibial nail length

## Abstract

Objective: Although various methods have been identified for the appropriate tibial nail length, there are disadvantages such as extra time, radiation exposure, and additional cost. We aimed to predict the ideal length of the nail according to the patient's height and shoe size in the preoperative period to eliminate these disadvantages.

Materials and Methods: Between January 2018 and August 2023, the height and shoe size data of 71 patients who were diagnosed with tibial shaft fracture and had tibia intramedullary nail fixation were determined. A linear regression model was applied to the normally distributed data. The correlation coefficient between the variables was evaluated according to the Pearson correlation coefficient.

Results: While 1 1-centimeter (cm) increase in patient height causes an increase of 3.2 millimeters (mm) in nail length, it was found that it alone contributed by 89% to predicting the ideal nail length (p<0.001). An increase of 1 unit in shoe size (EU) causes an increase of 9 mm in nail length; its contribution to predicting the ideal nail length alone is 84% (p<0.001). In the evaluation of the patient's height and shoe size together, it was observed that they jointly contributed 91% to the accurate estimation of the nail length (p<0.001).

Conclusion: It may be risky to determine the tibial nail length only by shoe size or the patient's height. However, the correlation between them is strong. Therefore, by estimating nail lengths before the operation, the ideal nail length can be found by making fewer fluoroscopy checks during the operation.

## Introduction

Traffic accidents fall from heights, or contact sports injuries often result in long bone fractures, mainly tibia fractures [1]. Today, intramedullary nail (IMN) fixation is known as the gold standard treatment method in the treatment of fractures in the diaphyseal region of the tibia, allowing early weight-bearing and medullary fixation [2]. One of the important elements in fracture fixation with IMN is to determine the appropriate length of the nail in the preoperative period. A nail placed shorter than the ideal length may cause reduction loss and implant failure, while a longer nail may lead to anterior knee pain, patellar tendon problems, and joint surface fractures [3]. Although various anthropometric measurements have been described to predict the ideal length of these nails preoperatively [4-6], there is still no consensus on the measurement methods, and this issue continues to attract the attention of researchers.

In the literature, some methods have been described for measuring the ideal nail length during the perioperative period. Examples of these methods include the use of a radiographic ruler, the use of a control guide wire, or checking the opposite leg with a nail under the scope [4,7,8]. However, these applications prolong the surgical time, cause the patient and the surgical team to be exposed to more radiation, and lead to an increase in costs due to unnecessary opening of implants [9,10]. In addition, there are publications in the literature stating that these methods are not always reliable [10,11].

Studies to determine the ideal implant size in preoperative preparation in locomotor system surgery continue to attract the attention of researchers. Examples of these are the studies reporting that the shoe size of the individual is positively correlated with the size of the femoral and tibial components in the knee prosthesis [12,13]. Considering this correlation for the locomotor system, there may be a connection between shoe size and the length of the IMN. In our study, if we can determine the ideal length of the IMN in the preoperative period based on the patient's height and shoe size, we can shorten the surgical time, reduce radiation exposure, and decrease many complications, especially nonunion.

## Materials and methods

For this retrospective study, approval was obtained from the ethics committee of Recep Tayyip Erdogan University Faculty of Medicine, with the decision number 2023/212 and the date September 14, 2023. Between January 2018 and August 2023 at the Recep Tayyip Erdogan University Orthopedics and Traumatology clinic, archival data of 83 patients aged 18 years and over who underwent surgical treatment with cannulated IMN the diagnosis of tibial shaft fractures were analyzed. Seventy-one patients who met the inclusion criteria of the ideal length IMN, whose archival data and postoperative tibia radiograph images were accessed, were included in the study. Successful union was achieved in all patients included in the study. Patients under the age of 18, those not treated with IMN at the ideal length, those unwilling to participate in the study, and those with a history of lower extremity fractures, lower extremity deformities, bone dysplasia, and congenital anomalies such as dwarfism or achondroplasia were excluded from the study. Patients who may have caused height loss after IMN surgery, such as lower limb pathology or spinal problems, were also not included in the study. The length of the tibial nail was obtained from the records in the hospital archive system. End cup lengths were also added to this nail length. Patients were contacted using the phone numbers from the archive records to provide information about the study. Height and shoe sizes were learned and recorded. This study was conducted in accordance with the principles of the Declaration of Helsinki.

In the literature, it is mentioned that to understand if the tibia nail is placed at the ideal length, it should be approximately 5-10 mm distal to the proximal tibial joint surface and 10-20 mm proximal to the distal tibial joint surface [5,6]. Patients with implanted nails outside these distances were also not included in the study.

Before the study, power analysis was performed using G-power to determine the number of samples. Using the mean and standard deviation data of the tibial nail length and patient shoe size, the effect power was calculated as 0.34. When alpha was 0.05 and 1-beta was 0.8, the number of total sample size was determined as 63.

Statistical analysis was conducted using the Statistical Package for the Social Sciences version 22.0 (IBM Corp., Armonk, NY, USA). As a statistical analysis, a linear regression model was applied since it was seen that the data showed normal distribution in the data preprocessing. We used Pearson’s correlation analysis to evaluate the relationship among numerical variables. We set statistical significance at p<0.05. The correlation analysis and scatter plots were made using Jamovi 2.4.6. (The Jamovi project - 2023).

## Results

Of the 71 patients, 53 (74.6%) were male and 18 (25.4%) were female. The mean age was 35.4 (±16.5) years. The height of the patients was between 160 and 185 (172±8.2) cm, and their shoe size (EU) was between 38 and 45 (41.5±2.4). The lengths of the tibial nails used ranged from 280 to 380 (324.4±25.6) mm (Table 1).

**Table 1:** Height and shoe size according to tibial nail lengths

Nail length (mm)	Number of patients	Height (cm)	Shoe size (EU)
280	3	161.3	38
300	18	165.9	38.83
320	18	171	39.56
330	9	171.4	42.33
340	13	176.7	42.77
350	1	183	44
360	6	181	43.67
370	1	182	44
380	2	184	44.5

**Figure 1:**
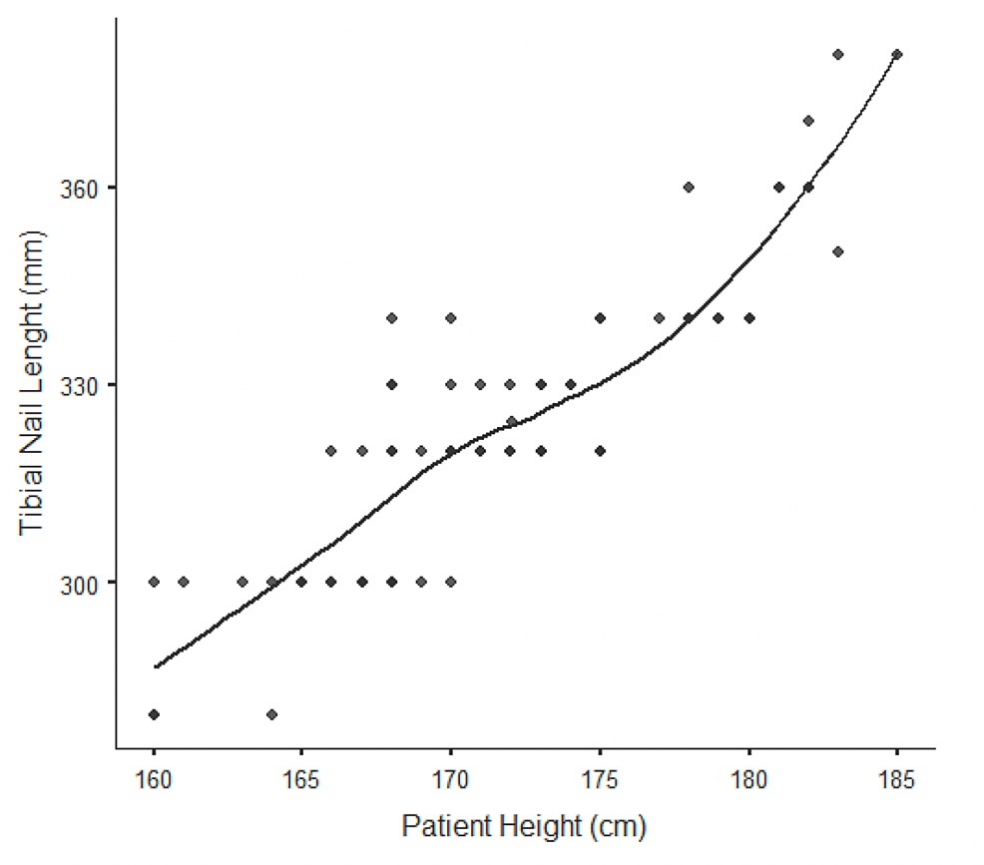
Relationship between patient height and tibial nail length A 1-cm increase in patient height leads to an average increase of 3.2 mm in tibial nail length. It was observed that the patient's height alone contributed 89% to predicting the ideal tibial nail length (Pearson’s r: 0.893), (p<0.001).

**Figure 2:**
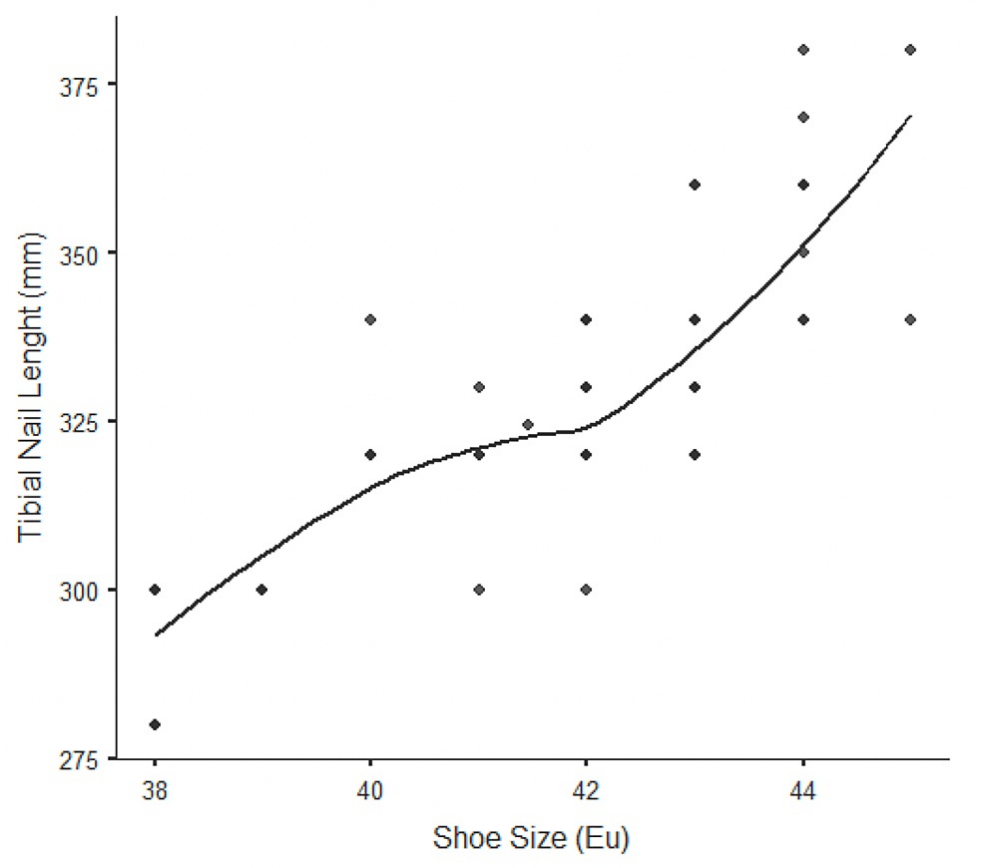
The relationship between shoe size and tibial nail length A 1-unit increase in shoe size (EU) leads to an average increase of 9 mm in tibial nail length. It was observed that the shoe size alone contributed 84% to predicting the ideal tibial nail length (Pearson’s r: 0.841), (p<0.001).

In the examination of the effect of patient height on nail length, a 1-cm increase in patient height leads to a 3.2-mm increase in nail length (Figure 1). It was observed that patient height alone contributed by 89% to predicting ideal nail length (p<0.001). Likewise, in the examination of the effect of shoe size alone on nail length, a 1-unit increase in shoe size (EU) causes a 9 mm increase in nail length (Figure 2). It was observed that shoe size alone contributed by 84% to predicting the ideal nail length (p<0.001). In the evaluation of the patient's height and shoe size together, it was seen that it contributed by 91% to the probability of estimating the nail length correctly (p<0.001).

## Discussion

In our study, we present an easy and alternative evaluation method that provides a highly accurate preoperative estimation of the ideal length of the intramedullary tibial nail, which is the gold standard treatment method in tibial shaft fractures. In our study, in which we investigated the predictability of the ideal tibial nail length in the preoperative period, patient height alone provides an accuracy rate of 89%, while shoe size alone provides an accuracy rate of 84%. As shown in Figure 1, a 1-cm in patient height leads to an average increase of 3.2 mm in tibial nail length. Also, as shown in Figure 2, a 1-unit increase in shoe size (EU) leads to an average increase of 9 mm in tibial nail length. In our study, in which the patient's height and shoe size were evaluated together for the first time, this rate reached a high value of 91%. 

Although there are studies in the literature with anthropometric methods [4,14,15] such as measuring the distance between the olecranon-fifth metacarpal head or with different measurement methods such as measuring the opposite extremity on radiography [3,9,10], there are limited studies evaluating the actual length of nails used with ideal length. Moreover, it is possible to come across studies that do not recommend the use of this anthropometric measurement due to its very low accuracy (51%) [4]. It can also be evaluated as an alternative to this measurement in patients with upper extremity deformities. There is unnecessary extra radiation exposure in radiographic measurements. There are publications stating that anatomical measurements are not sufficient to predict the length of the tibial nail [3].

In the study of Pearson et al., which showed similar results to our study, the accuracy rate in estimating the femoral IMN length according to patient height was 86% for antegrade nails and 88% for retrograde nails [16]. In addition, in another study evaluating the height alone, the accuracy rate in estimating the nail length was shown to be 82% [1]. These studies demonstrate that it is not feasible to accurately predict the correct nail length for a group of patients solely based on height. Therefore, this estimation can be strengthened with a second parameter such as shoe size.

Although there are few studies in the literature showing that the ideal IMN length can be predicted according to patient height, we have not found any study showing that shoe size provides a similar high-accuracy estimation of nail length. Especially in patients with severe vertebral alignment disorders, those with bilateral hip dislocations at a high level, patients with shortening due to past pelvis or bilateral lower extremity fractures, those with advanced contractures in the hip-knee joints, or patients with rhizomelic dwarfism such as achondroplasia, we believe that predicting tibia nail length solely based on height may lead to incorrect results. In such patients, the ideal nail length can be predicted with a high degree of accuracy by making use of the shoe size proportional to the height (Table 1). Shoe size measurement is a good alternative to height for this estimation, especially in the patients with lower extremity contractures or deformities for whom erroneous height measurement can be made.

In the studies, the ratio of foot length to height is approximately 15% [17]. However, it should be noted that in taller individuals, this ratio can vary, so predictions based solely on shoe size should not be made for these patients. Although this positive relationship between shoe size and tibial nail length is the first case in the literature, it has been shown that this measurement is a significantly useful determinant in predicting the size of the femoral and tibial components in total knee prosthesis [12,18,19].

Our study has some limitations. Gender distribution was not balanced in our study, as tibial shaft fractures are more common in males [20,21]. New studies conducted in a larger population where the number of female participants is sufficient may yield more beneficial results in predicting the length of the intramedullary tibia nail. In addition, studies on people who wear the same style shoes, such as military personnel, may be more useful to show the relationship between shoe size and nail length in order to obtain more reliable results in questioning the shoe size of the patients, since not everyone wears the same style of shoes.

## Conclusions

Both patient's height and shoe size were strongly correlated with tibial nail length. Especially together, can be used as a valuable determinant in preoperative nail length planning for intramedullary tibia nailing. People with the same shoe size or the same height may have different ideal tibia nail lengths. But all of these were lengths close to each other. When the ideal tibia intramedullary nail length, patient height, and shoe size are evaluated together, it can be predicted with a high rate of 91% in the pre-operative period. While a 1-cm increase in patient height causes an increase of 3.2 mm in ideal nail length, an increase of 1 unit in shoe size (EU) causes an increase of 9 mm in nail length. According to the linear regression model, this measurement will result in much less effort and time compared to traditional methods such as the use of radiographic calipers, control guide wires, or checking the nail's alignment with the contralateral leg under fluoroscopy during the perioperative period, while also reducing radiation exposure.
